# Characterization of telomere length in Agerolese cattle breed, correlating blood and milk samples

**DOI:** 10.1111/age.13227

**Published:** 2022-07-01

**Authors:** Alessandra Iannuzzi, Sara Albarella, Pietro Parma, Giacomo Galdiero, Emanuele D'Anza, Ramona Pistucci, Vincenzo Peretti, Francesca Ciotola

**Affiliations:** ^1^ Institute for Animal Production System in Mediterranean Environment National Research Council Naples Italy; ^2^ Department of Veterinary Medicine and Animal Production University of Naples Federico II Naples Italy; ^3^ Department of Agricultural and Environmental Sciences University of Milan Milan Italy; ^4^ Division of Endocrinology, Department of Clinical Medicine and Surgery, Unity of Andrology and Medicine of Reproduction and Male and Female Sexuality (FERTISEXCARES) University of Naples Federico II Naples Italy

**Keywords:** Agerolese cattle, animal welfare biomarker, blood and milk samples, quantitative PCR, telomere length

## Abstract

Studies into telomere length in cattle are relatively recent and have focused mainly on the Holstein Friesian cattle breed, making it arduous to evaluate the correlation with ageing due to the early age of culling in this breed. Telomere length provides information about the productive lifespan and the quality of farm management, complying with the ‘One Health’ approach. This study evaluated telomere length in Agerolese cattle, an autochthonous dairy breed characterized by a long productive lifespan (13 years). Multiplex quantitative PCR estimated telomere length in DNA extracted from blood and milk matrices. Interestingly, the results showed longer telomeres in Agerolese (compared to the Holstein Friesian cattle control group), with a negative correlation between telomere length and increasing age and a synchronous trend between blood and milk samples, with a positive correlation between them.

Telomeres are specialized nucleoprotein structures that are localized at the end of chromosomes and formed by thousands of noncoding repetitive sequences of DNA associated with six proteins, the shelterin complex. Recent studies have related telomere length (TL) to longevity (Brown et al., 2012) and welfare (Bateson, 2016; Beloor et al., 2010) in farm animals. In cattle, studies into TL have focused mainly on the Holstein Friesian breed (HFR) (Ilska‐Warner et al., 2019; Seeker et al., 2018b) and the blood matrix (Seeker et al., 2018a), providing little information about their ratio in other cattle breeds (Tilesi et al., 2010) and different biological matrix (Iannuzzi et al., 2020; Laubenthal et al., 2016).

We focused our study on Agerolese (AGR) cattle, an autochthonous Italian breed characterized by good productive longevity, to better understand the TL correlation with breed and age (age at culling between 8 and 13 years). The environment, the type of breeding, and the mild zootechnical selective pressure are probably the basis of the genome stability of this breed (Ciotola et al., 2005; Iannuzzi et al., 2015).

The objectives of this study were to evaluate: (1) the TL correlation between blood and milk samples comparing relative leukocyte telomere length (RLTL) and relative milk cell telomere length (RMCTL) for the first time; and (2) TL in AGR cattle, comparing it to another breed (HFR) at the same age (24–78 months) and physiological stage (first lactation).

The HFR was chosen for comparison because it is the most studied and widespread breed of cattle. The study was approved by the Ethical Animal Care and Use Committee of the University of Naples Federico II (Prot. Nr. PG/2019/0104896), with informed consent from the cows' owners. Healthy cattle were split into two groups according to their breed: 49 AGR (25–78 months) and 52 HFR (26–73 months). Milk (somatic cell [SC] count <200,000 cells/ml) and blood samples were collected simultaneously during the spring season at the same lactation stage (100 days postpartum). We also included a group of 42 older AGR (AGRe, 96–158 months) for comparison with the AGR group (Supporting Information) to evaluate TL in older cattle. According to their manufacturer's protocol, genomic DNA was isolated from blood (leukocytes) samples using the Wizard Genomic DNA Purification Kit (Promega). In contrast, genomic DNA from milk (SCs) samples was isolated using the QIAamp Fast DNA Stool Mini Kit (QIAGEN). DNA yield and purity were measured on a NanoDrop ND‐1000 spectrophotometer (Thermo Scientific) for each sample. Before the quantitative polymerase chain reaction (qPCR) analysis, all the genomic DNA samples were diluted at 10 ng/μl in nuclease‐free water and stored at −20°C.

DNA showed the following requirements: yield >30 ng/μl, 260/280 ratio >1.7, and 260/230 ratio >1.8. All methods for monochrome multiplex qPCR followed the guidelines and regulations for qPCR experiments (Bustin et al., 2009) and TL measurement using qPCR approaches (Lindrose et al., 2021; Nettle et al., 2019). The total reaction mix per well (20 μl) contained SYBR Green Supermix (Biorad), genomic DNA, and forward and reverse primer sequences for telomere and β‐globin (defined as SCG), following Cawthon (2009). Each sample was assayed in triplicates (intra‐assay) in three different runs (inter‐assay) with negative control (NTC), and a standard curve for each primer evaluated the amplification efficiency and linearity. The quantification cycle (Cq) values presented a standard deviation (SD) <0.25, and relative TL was calculated using the ΔΔCt method with the Pfaffl correction (Pfaffl, 2001). More details are reported in the Supporting Information section.

IBM SPSS analyzed the results for the Windows software package version 22.0 (SPSS Inc.). Skewness test, kurtosis test, *Z* value and Shapiro test were performed to verify parameters distribution (parametric/non‐parametric) and data are presented as medians and interquartile ranges. Since the data were distributed in a nonparametric way, we compared independent groups using the nonparametric Mann–Whitney test for continuous variables. Spearman's test was used to assess correlation among RLTL, RMCTL, and age (Figure 1, Figures S1 and S2). Statistical significance was set at *p* ≤ 0.05.

**FIGURE 1:**
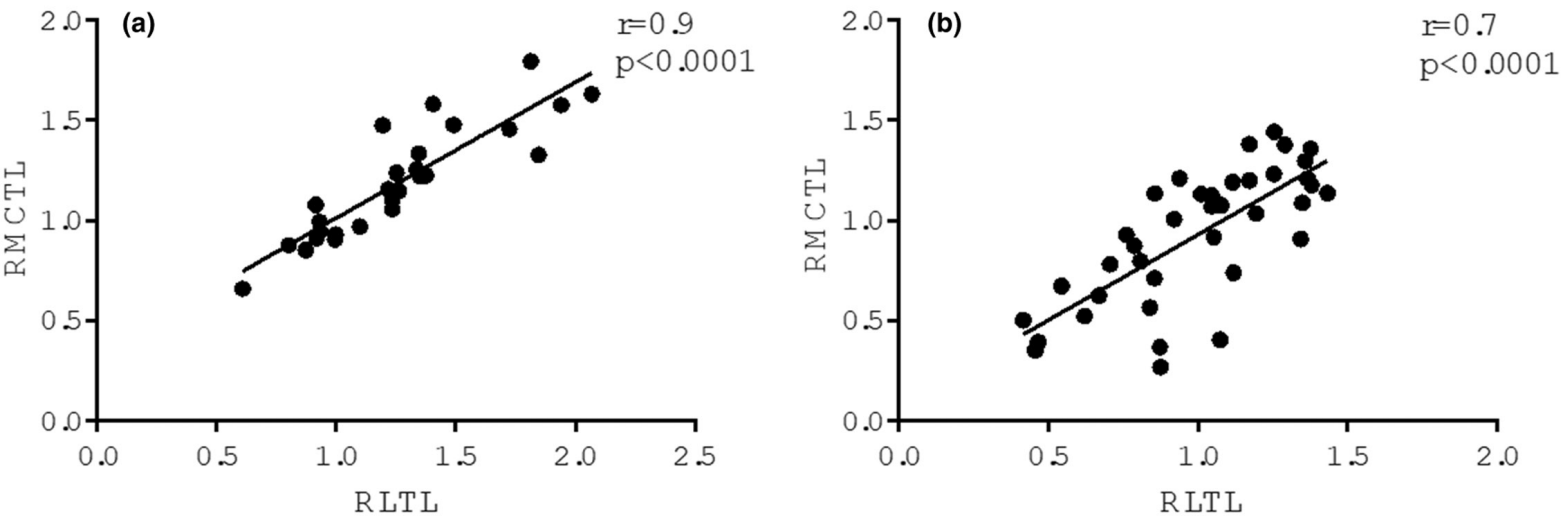
Relative milk cell telomere length (RMCTL) and relative leukocyte telomere length (RLTL) correlations in the Agerolese (a) and Holstein Friesian (b) groups at the first lactation. Spearman's correlation analysis shows a significant strong positive correlation between RMCTL vs. RLTL in Agerolese (*r* = 0.9; *p* < 0.0001) and in Holstein Friesian (*r* = 0.7; *p* < 0.0001)

Blood and milk matrices were compared for the first time, both in the AGR and HFR groups; a strong positive correlation between RMCTL and RLTL was found, both in groups at the first lactation (AGR; *r* = 0.9; *p* < 0.0001; Figure 1a. HFR; *r* = 0.7; *p* < 0.0001; Figure 1b) and in the two entire groups (AGR; *r* = 0.9; *p* < 0.0001; Figure S1a. HFR; *r* = 0.7; *p* < 0.0001; Figure S1b). One probable reason for this result could be the kind of cells present in both matrices. Milk SCs (lymphocytes, exfoliated epithelial cells, neutrophils, and macrophages) are usually present at low levels, varying with the health status and breed of the animal. SCs in milk are a typical physiological finding necessary for the regeneration of normal epithelia. The mammary epithelial cells are lined by blood vessels absorbing several milk precursors from blood and they are responsible for milk synthesis and release into the alveolar lumen (Alhussien & Dang, 2018). For this reason, any TL variation in the blood is also reflected in milk, as shown in our data (Table 1 and Table S1). In this way, the milk sample represents a suitable alternative, which is easier and less stressful to collect than blood samples from dairy animals.

**TABLE 1:** Comparison analysis of the two groups in the first lactation (Agerolese [AGR] and Holstein Friesian [HFR]) for age in months, relative leukocyte telomere length (RLTL), and relative milk cells telomere length (RMCTL)

Median (5th–95th IQR)				
Group	*n*	Age	RLTL	RMCTL
AGR	27	36 (25–64.6)	1.2 (0.7–2.0)^a^	1.2 (0.7–1.7)^a^
HFR	38	37 (26.9–69.2)	1.0 (0.4–1.4)^b^	1.0 (0.3–1.4)^b^

*Note*: Values are expressed as median and interquartile range (IQR). The comparison analysis shows a statistical significance between HFR vs. AGR (*p* < 0.01) in RLTL and RMCTL. A significant difference was found ^ab^ *p* < 0.01.

The TL comparison analysis between the AGR and HFR groups showed a statistically significant difference. RLTL and RMCTL always resulted higher in AGR than in the HFR groups at the first lactation (RLTL *p* < 0.01; RMCTL *p* < 0.01; Table 1) and also when the two entire groups were compared (RLTL *p* < 0.05; RMCTL *p* < 0.01; Table S1). Tilesi et al. (2010) studied TL in two Italian cattle breeds (Chianina and Maremmana) used for meat production and characterized by high longevity and range breeding; longer telomeres were observed in Maremmana than in Chianina. Interestingly, the selection history of Maremmana (the breed with longer telomeres) is much more similar to that of AGR (reblood with individuals of other breeds for the heads' contraction in recent years) than HFR. Furthermore, the AGR breed is also characterized by high genome stability, probably due to the environment and mild zootechnical selective pressure (Ciotola et al., 2005). Both AGR and HFR were healthy, sampled simultaneously, and reared in the same area and under similar conditions, confirming that telomeres are also determined by heritable quantitative traits derived from both genetic and epigenetic mechanisms (Eisenberg & Kuzawa, 2018). Furthermore, we noticed that relative TL in AGR is naturally longer than in HFR at the same age and lactation stages (Table 1).

The correlations of RLTL were negative regarding the age of the animal (reported in months) in the AGR group compared with AGRe, showing a significant negative regular correlation between RLTL vs. age in months (*r* = −0.5; *p* < 0.0001; Figure S2). Before our study, only Miyashita et al. (2002) studied TL, by terminal restriction fragment, in leukocytes from 50 Japanese black cattle sampled one time (age range 0–18 years), showing the decline in telomere lengths with age. In line with Miyashita et al. (2022), our results demonstrated that RLTL reported an age‐dependent decline (Figure S2) not linked to a specific pathology, as reported in humans, but to senescence. This is probably due to better maintenance of telomerase expression and the lack of age‐related diseases (including cancer, atherosclerosis, autoimmune disorders, obesity, chronic obstructive pulmonary disease, diabetes, hematological disorders, and neurodegenerative diseases) often associated with the increased speed of telomere shortening in elderly humans (Kordinas et al., 2016). Sometimes, it is difficult to separate the effects of chronic and/or age‐related diseases from normal ageing. For this reason, TL can be considered a relevant biomarker of farm animals' general state, revealing potentially risky diseases and environmental stress (Iannuzzi et al., 2019; Seeker et al., 2021) due to the lack of age‐related pathologies.

Still, we observe that the TL in the HFR breed, whose median age at culling is 6 years, is significantly lower than in the AGR breed, supporting the hypothesis that a higher TL may indicate a longer productive lifespan.

To summarize, our results revealed a higher TL in a long‐living autochthonous cattle breed (Agerolese). Since TL heritability has been shown (Seeker et al., 2018a), and a longer TL is observed in a cattle breed with a longer productive lifespan, the inclusion of this parameter in the breeding selection plans could be interesting. Moreover, this study shows that milk, a biological matrix that is easier to collect from dairy animals than blood, can be used to evaluate telomere length.

## CONFLICT OF INTEREST

The authors declare that no competing interests exist.

## Supporting information


Figure S1
Figure S2
Table S1

## Data Availability

The data that support the findings of this study are available from the corresponding author, [AI], upon reasonable request.

## References

[age13227-bib-0001] Alhussien, M.N. & Dang, A.K. (2018) Milk somatic cells, factors influencing their release, future prospects, and practical utility in dairy animals: an overview. Vet World, 11, 562–577. 29915493 10.14202/vetworld.2018.562-577 PMC5993762

[age13227-bib-0002] Bateson, M. (2016) Cumulative stress in research animals: telomere attrition as a biomarker in a welfare context? BioEssays, 38, 201–212. 26645576 10.1002/bies.201500127 PMC4737400

[age13227-bib-0003] Beloor, J. , Kang, H.K. , Kim, Y.J. , Subramani, V.K. , Jang, I.S. , Sohn, S.H. et al. (2010) The effect of stocking density on stress related genes and telomeric length in broiler chickens. Asian‐Australasian Journal of Animal Sciences, 23, 437–443.

[age13227-bib-0004] Brown, D.E. , Dechow, C.D. , Liu, W.S. , Harvatine, K.J. & Ott, T.L. (2012) Hot topic: association of telomere length with age, herd, and culling in lactating Holsteins. Journal of Dairy Science, 95, 6384–6387. 22981568 10.3168/jds.2012-5593

[age13227-bib-0005] Bustin, S.A. , Benes, V. , Garson, J.A. , Hellemans, J. , Huggett, J. , Kubista, M. et al. (2009) The MIQE guidelines: minimum information for publication of quantitative real‐time PCR experiments. Clinical Chemistry, 55, 611–622. 19246619 10.1373/clinchem.2008.112797

[age13227-bib-0006] Cawthon, R.M. (2009) Telomere length measurement by a novel monochrome multiplex quantitative PCR method. Nucleic Acids Research, 37, e21. 19129229 10.1093/nar/gkn1027 PMC2647324

[age13227-bib-0007] Ciotola, F. , Peretti, V. , Di Meo, G.P. , Perucatti, A. , Iannuzzi, L. & Barbieri, V. (2005) Sister chromatid exchanges (SCE) in the Agerolese cattle population. Veterinary Research Communications, 29(Suppl. 2), 359–361. 16244994 10.1007/s11259-005-0081-6

[age13227-bib-0008] Eisenberg, D.T.A. & Kuzawa, C.W. (2018) The paternal age at conception effect on offspring telomere length: mechanistic, comparative and adaptive perspectives. Philosophical Transactions of the Royal Society of London. Series B, Biological Sciences, 373(1741), 20160442. 29335366 10.1098/rstb.2016.0442 PMC5784062

[age13227-bib-0009] Iannuzzi, A. , Della, V.G. , Russo, M. , Longobardi, V. , Albero, G. , De Canditiis, C. et al. (2020) Evaluation of bovine sperm telomere length and association with semen quality. Theriogenology, 158, 227–232. 32980685 10.1016/j.theriogenology.2020.09.019

[age13227-bib-0010] Iannuzzi, A. , Genualdo, V. , Perucatti, A. , Pauciullo, A. , Varricchio, G. , Incarnato, D. et al. (2015) Fatal outcome in a newborn calf associated with partial trisomy 25q and partial monosomy 11q, 60,XX,der(11)t(11;25)(q11;q14 approximately 21). Cytogenetic and Genome Research, 146, 222–229. 26337016 10.1159/000438973

[age13227-bib-0011] Iannuzzi, A.P.A. , Genualdo, V. , Rossetti, C. , Iorio, C. , Caputi, J.A. , Giannico, F. et al. (2019) Cytogenetic and genomic investigations in river buffaloes raised in farms located in urban and rural areas of Campania region (southern‐Italy). Annals of Carcinogenesis, 4(1), 1020.

[age13227-bib-0012] Ilska‐Warner, J.J. , Psifidi, A. , Seeker, L.A. , Wilbourn, R.V. , Underwood, S.L. , Fairlie, J. et al. (2019) The genetic architecture of bovine telomere length in early life and association with animal fitness. Frontiers in Genetics, 10, 1048. 31749836 10.3389/fgene.2019.01048 PMC6843005

[age13227-bib-0013] Kordinas, V. , Ioannidis, A. & Chatzipanagiotou, S. (2016) The telomere/telomerase system in chronic inflammatory diseases. Cause or effect? Genes (Basel), 7(9), 60. 10.3390/genes7090060 PMC5042391 27598205

[age13227-bib-0014] Laubenthal, L. , Hoelker, M. , Frahm, J. , Danicke, S. , Gerlach, K. , Sudekum, K.H. et al. (2016) Short communication: telomere lengths in different tissues of dairy cows during early and late lactation. Journal of Dairy Science, 99, 4881–4885. 26995138 10.3168/jds.2015-10095

[age13227-bib-0015] Lindrose, A.R. , McLester‐Davis, L.W.Y. , Tristano, R.I. , Kataria, L. , Gadalla, S.M. , Eisenberg, D.T.A. et al. (2021) Method comparison studies of telomere length measurement using qPCR approaches: a critical appraisal of the literature. PLoS One, 16, e0245582. 33471860 10.1371/journal.pone.0245582 PMC7817045

[age13227-bib-0022] Miyashita, N. , Shiga, K. , Yonai, M. , Kaneyama, K. , Kobayashi, S. , Kojima, T. et al. (2002) Remarkable differences in telomere lengths among cloned cattle derived from different cell types. Biology Reproduction, 66, 1649–55. 10.1095/biolreprod66.6.1649 12021043

[age13227-bib-0016] Nettle, D. , Seeker, L. , Nussey, D. , Froy, H. & Bateson, M. (2019) Consequences of measurement error in qPCR telomere data: a simulation study. PLoS One, 14, e0216118. 31042766 10.1371/journal.pone.0216118 PMC6493763

[age13227-bib-0017] Pfaffl, M.W. (2001) A new mathematical model for relative quantification in real‐time RT‐PCR. Nucleic Acids Research, 29, 45e–445e. 10.1093/nar/29.9.e45 PMC55695 11328886

[age13227-bib-0018] Seeker, L.A. , Ilska, J.J. , Psifidi, A. , Wilbourn, R.V. , Underwood, S.L. , Fairlie, J. et al. (2018a) Longitudinal changes in telomere length and associated genetic parameters in dairy cattle analysed using random regression models. PLoS One, 13, e0192864. 29438415 10.1371/journal.pone.0192864 PMC5811042

[age13227-bib-0019] Seeker, L.A. , Ilska, J.J. , Psifidi, A. , Wilbourn, R.V. , Underwood, S.L. , Fairlie, J. et al. (2018b) Bovine telomere dynamics and the association between telomere length and productive lifespan. Scientific Reports, 8, 12748. 30143784 10.1038/s41598-018-31185-z PMC6109064

[age13227-bib-0020] Seeker, L.A. , Underwood, S.L. , Wilbourn, R.V. , Dorrens, J. , Froy, H. , Holland, R. et al. (2021) Telomere attrition rates are associated with weather conditions and predict productive lifespan in dairy cattle. Scientific Reports, 11, 5589. 33692400 10.1038/s41598-021-84984-2 PMC7970942

[age13227-bib-0021] Tilesi, F. , Domenico, E.G.D. , Pariset, L. , Bosco, L. , Willems, D. , Valentini, A. et al. (2010) Telomere length diversity in cattle breeds. Diversity, 2, 1118–1129.

