# Supplementary material for: Characterization of telomere length in Agerolese cattle breed, correlating blood and milk samples
Source: Anim Genet. 2022 Jul 1;53(5):676–9. doi: 10.1111/age.13227 (PMC9544343; doi:10.1111/age.13227)
Supplement: Supplementary file 1 — Figure S1 Figure S2 Table S1 [file AGE-53-676-s001.docx]

**SUPPORTING INFORMATION**

**Study population and sampling**

All dairy cows involved in the study were reared in Sorrento Peninsula and fed with the same lactation diet. AGR cows were enrolled in the Birth Register (285 animals) and raised in the “Provolone del Monaco” PDO cheese production area. We have also included 41 older AGRe DNA samples from our AGR DNA bank (blood samples) in this study. Whole blood samples were taken by venipuncture using EDTA as an anticoagulant and stored at -20°C at least one week before DNA extraction. Milk samples were taken during the mechanical milking using sterile tubes and placed at 4°C for maximum 7 hours before DNA extraction.

**Multiplex qPCR method**

qPCR was performed using a CFX RT-PCR System (Biorad) with samples randomly allocated on a qPCR 96 well plate (Hard-Shell PCR Plates-Biorad). Both the telomere and the reference gene (single copy gene-SCG) reactions were performed in the same wells (multiplex qPCR) after having tested them individually (monoplex qPCR) on 10 random samples. One calibrator or golden sample (from blood) was repeated on each plate and included in the calculation of relative TL (RTL) to compare its measurements from different qPCR plates. The reaction total mix per well (20 µl) contained 10μl iTaq Universal SYBR Green Supermix (Biorad), 30 ng of genomic DNA, and forward and reverse primer sequences for telomere (500 mM) and beta-globin (250 mM - defined as SCG), following Brown et al. indications ([Brown et al. 2012](#_ENREF_6)). Each sample was assayed in triplicates in three different runs with negative control (NTC). The 73°C read provides the telomere amplification's quantification cycle (Cq) value. In comparison, the 83°C read provides the Cq value for the amplification of the SCG template (at this temperature, no signal is detected from the telomere amplification product because it is completely melted). A standard curve for each primer was evaluated in each assay to assess amplification efficiency and linearity. Four concentrations of cattle genomic DNA, obtained from four samples, were prepared by four-fold serial dilutions, starting with 120 ng/μl.

**qPCR efficiency and quality control**

The Biorad CFX Maestro software was used for a baseline correction of the calculation of the plate and reaction-specific qPCR efficiency and the analysis of Cq values. The qPCR efficiency (E) was 95,2% for the telomere and 97,4% for SCG. Cq values were calculated by setting thresholds, for all plates, within the window of linearity. The intra-assay precision was determined in three repeats within one run to confirm the accuracy of qPCR. The Cq values with a standard deviation (SD) of greater than 0.25 among triplicates were repeated a maximum of three times to satisfy the qPCR quality control requirements. The inter-assay variation was investigated to confirm the reproducibility of qPCR, repeating the measurements in triplicate on three different runs on consecutive days and showing an SD less than 0.25 among the means of the same sample. Less than 2% of samples failed the qPCR quality control requirements and were excluded from the analysis.

**Table S1.** The comparison analysis of the two entire groups (AGR and HFR) for months-old age, RLTL, and RMCTL. Values expressed as median and IQR. The comparison analysis shows a statistical significance between HFR vs. AGR in RLTL (p<0.05) and RMCTL (p<0.01). A significant difference was found ^ab^p < 0.05; ^cd^p < 0.01.

| **MEDIAN (5°-95° IQR)** | | | | |
| --- | --- | --- | --- | --- |
| **Group** | ***n*** | **Age** | **RLTL** | **RMCTL** |
| **AGR** | 48 | 46.5 (25-75) | 1.1 (0.8-1.9)^a^ | 1.1 (0.9-1.6)^c^ |
| **HFR** | 51 | 44 (27.6-72) | 1.0 (0.4-1.4)^b^ | 1.0 (0.4-1.4)^d^ |


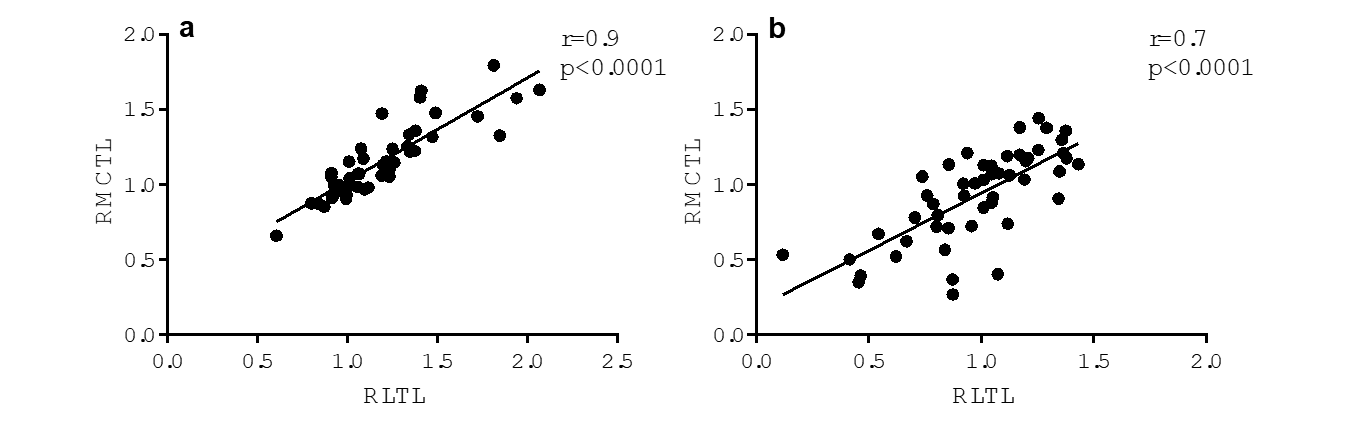


**Figure S1.** RMCTL (relative somatic cells telomere length) and RLTL (relative leukocyte telomere length) correlations in the AGR (a) and HFR (b) entire groups. Spearman’s correlation analysis shows a significant strong positive correlation between RMCTL vs. RLTL in AGR (r = 0.9; p < 0.0001) and HFR (r = 0.7; p < 0.0001).

**Figure S2.** RLTL (relative leukocyte telomere length) and age (in months) correlations in the AGR and AGRe groups. Spearman’s correlation analysis shows a significant negative regular correlation in the AGR (r = -0.5; p < 0.0001) group.
